# Beyond Nuclear Ribosomal DNA Sequences: Evolution, Taxonomy, and Closest Known Saprobic Relatives of Powdery Mildew Fungi (*Erysiphaceae*) Inferred From Their First Comprehensive Genome-Scale Phylogenetic Analyses

**DOI:** 10.3389/fmicb.2022.903024

**Published:** 2022-06-09

**Authors:** Niloofar Vaghefi, Stefan Kusch, Márk Z. Németh, Diána Seress, Uwe Braun, Susumu Takamatsu, Ralph Panstruga, Levente Kiss

**Affiliations:** ^1^Centre for Crop Health, Institute for Life Sciences and the Environment, University of Southern Queensland, Toowoomba, QLD, Australia; ^2^Faculty of Veterinary and Agricultural Sciences, University of Melbourne, Parkville, VIC, Australia; ^3^Unit of Plant Molecular Cell Biology, Institute for Biology I, RWTH Aachen University, Aachen, Germany; ^4^Plant Protection Institute, Centre for Agricultural Research, Eötvös Loránd Research Network, Budapest, Hungary; ^5^Department of Geobotany and Botanical Garden, Herbarium, Institute for Biology, Martin Luther University of Halle-Wittenberg, Halle (Saale), Germany; ^6^Laboratory of Plant Pathology, Faculty of Bioresources, Mie University, Tsu, Japan; ^7^Centre for Research and Development, Eszterházy Károly Catholic University, Eger, Hungary

**Keywords:** contaminated genomes, contaminating sequences, metagenomes, obligate biotrophs, phylogenomics, plant–microbe interactions, single-copy orthologs, whole-genome sequencing

## Abstract

Powdery mildew fungi (*Erysiphaceae*), common obligate biotrophic pathogens of many plants, including important agricultural and horticultural crops, represent a monophyletic lineage within the *Ascomycota*. Within the *Erysiphaceae*, molecular phylogenetic relationships and DNA-based species and genera delimitations were up to now mostly based on nuclear ribosomal DNA (nrDNA) phylogenies. This is the first comprehensive genome-scale phylogenetic analysis of this group using 751 single-copy orthologous sequences extracted from 24 selected powdery mildew genomes and 14 additional genomes from *Helotiales*, the fungal order that includes the *Erysiphaceae*. Representative genomes of all powdery mildew species with publicly available whole-genome sequencing (WGS) data that were of sufficient quality were included in the analyses. The 24 powdery mildew genomes included in the analysis represented 17 species belonging to eight out of 19 genera recognized within the *Erysiphaceae*. The epiphytic genera, all but one represented by multiple genomes, belonged each to distinct, well-supported lineages. Three hemiendophytic genera, each represented by a single genome, together formed the hemiendophytic lineage. Out of the 14 other taxa from the *Helotiales*, *Arachnopeziza araneosa*, a saprobic species, was the only taxon that grouped together with the 24 genome-sequenced powdery mildew fungi in a monophyletic clade. The close phylogenetic relationship between the *Erysiphaceae* and *Arachnopeziza* was revealed earlier by a phylogenomic study of the *Leotiomycetes*. Further analyses of powdery mildew and *Arachnopeziza* genomes may discover signatures of the evolutionary processes that have led to obligate biotrophy from a saprobic way of life. A separate phylogeny was produced using the 18S, 5.8S, and 28S nrDNA sequences of the same set of powdery mildew specimens and compared to the genome-scale phylogeny. The nrDNA phylogeny was largely congruent to the phylogeny produced using 751 orthologs. This part of the study has revealed multiple contamination and other quality issues in some powdery mildew genomes. We recommend that the presence of 28S, internal transcribed spacer (ITS), and 18S nrDNA sequences in powdery mildew WGS datasets that are identical to those determined by Sanger sequencing should be used to assess the quality of assemblies, in addition to the commonly used Benchmarking Universal Single-Copy Orthologs (BUSCO) values.

## Introduction

Nuclear ribosomal DNA (nrDNA) sequences, above all the internal transcribed spacer (ITS), the large subunit (LSU or 28S) and the small subunit (SSU or 18S) nrDNA sequences, have been at the epicenter of molecular identification and phylogenetic studies of all groups of fungi since the nascence of this research field in the late 1980s. According to Web of Science, the paper reporting the first universal primers to amplify the 18S and the ITS region of nrDNA in fungi (White et al., 1990) is probably the most cited publication in mycology, with over 40,000 citations to date. More than two decades later, a comprehensive, multi-laboratory comparison of different nuclear and mitochondrial DNA loci widely used in fungal phylogenetics concluded that the nrDNA ITS sequences are still the only reliable species DNA markers that can be used to infer phylogenies across all fungal groups (Schoch et al., 2012). In some intensively studied groups of fungi, multi-locus analyses based on specific sets of loci have already been established as the frameworks for phylogenetic analyses and molecular taxonomic studies (e.g., Marin-Felix et al., 2019; Vaghefi et al., 2020; Poudel et al., 2021); however, in most fungal groups, nrDNA sequences are still the sole basis for phylogenetic works (e.g., Crous et al., 2019, 2020, 2021).

An important group of ascomycetous plant pathogens, the *Erysiphaceae*, known as the powdery mildew fungi, is a good example for a large monophyletic lineage within which molecular phylogenetic relationships as well as DNA-based species and genera delimitations are mostly based on nrDNA phylogenies. The *Erysiphaceae* include more than 900 species belonging to 19 genera. All species are obligate biotrophic plant pathogens, i.e., they take up nutrients from living host plant tissues only (Hückelhoven and Panstruga, 2011) and cannot grow and produce spores for reproduction without being functionally connected to the infected and living host plant tissues. Altogether, powdery mildew fungi can colonize more than 10,000 dicot and monocot species in different parts of the world (Amano, 1986; Braun and Cook, 2012). Some powdery mildew species infect only a single host plant species or a few closely related hosts belonging to the same genus (e.g., Meeboon et al., 2017; Kiss et al., 2018). Others are known from many, only distantly related plants (e.g., Meeboon and Takamatsu, 2016; Braun et al., 2019; Kiss and Vaghefi, 2021; Young and Kiss, 2021). Finally, some host plants can be infected by more than one powdery mildew species, which sometimes belong to different genera (e.g., Takamatsu et al., 2007; Kiss et al., 2008; Desprez-Loustau et al., 2018; Kelly et al., 2021; Faticov et al., 2022). Some species have become invasive in different parts of the world (Kiss, 2005; Desprez-Loustau et al., 2010; Kiss et al., 2020). Important crops, including wheat, barley, grapevine, as well as fruit and vegetable species, are commonly colonized by diverse powdery mildew fungi (Glawe, 2008). Despite extensive research on their pathogenesis, epidemiology and control, these powdery mildew species remain amongst the economically most important plant pathogens in agriculture and horticulture worldwide due to the combined effect of costs of chemical crop protection measures and yield losses (Calonnec et al., 2004; Fondevilla and Rubiales, 2012; Fuller et al., 2014; Dunn and Gaynor, 2020). Others are well-known tree pathogens (Marçais and Desprez-Loustau, 2014; Demeter et al., 2021), and some have become model organisms in plant pathology research (Gadoury et al., 2012; Bindschedler et al., 2016; Kuhn et al., 2016) or in the study of wild plant pathosystems (Susi et al., 2015).

Before the era of DNA-based phylogenies, the generic phylogenetic concept in the *Erysiphaceae* was based on the morphological characteristics of the sexual morphs (teleomorphs), known as chasmothecia (formerly: cleistothecia; Braun, 1987, 1995). The complex geometries of the appendage tips of some chasmothecia have captured the attention and admiration of early mycologists and microscopists already in the 19th century (Hirata et al., 2000) and were useful in grouping powdery mildews in a few genera. Other characteristics of chasmothecia, such as the number and shape of asci and ascospores, have also been used to define the genera within the *Erysiphaceae*. Species within genera were mainly distinguished based on their host plants and the morphology of the sexual and asexual morphs (Braun, 1987, 1995). The early speculations on the evolution of powdery mildew fungi focused on chasmothecia, and presumed that species/genera with simple, mycelioid chasmothecial appendages were ancestral, and those with more complex appendages have appeared later during the evolution of the *Erysiphaceae* (Braun, 1987).

It was, therefore, surprising that the first phylogenetic analyses of powdery mildews based on nrDNA sequences did not support the classic, well-established generic concept of the *Erysiphaceae* (Takamatsu et al., 1998, 1999; Saenz and Taylor, 1999; Hirata et al., 2000; Mori et al., 2000a; Matsuda and Takamatsu, 2003). As one of the very first molecular phylogenetic analyses concluded, ‘appendage morphology does not always accurately reflect the phylogeny of the powdery mildews’ (Takamatsu et al., 1999). In fact, species were clearly grouped according to the morphological characteristics of their asexual morphs (anamorphs), irrespective of the morphology of their chasmothecia (Saenz and Taylor, 1999; Takamatsu, 2004). This has been confirmed by all nrDNA phylogenetic analyses conducted to date (e.g., Marmolejo et al., 2018; Bradshaw and Tobin, 2020; Kiss et al., 2020). The discovery that it is, in fact, the conidiogenesis and the morphology of the asexual morphs that mirror the phylogenetic relationships within the *Erysiphaceae* has triggered major changes in the taxonomy of powdery mildew fungi, especially at the genus level (Braun et al., 2002; Braun, 2011; Braun and Cook, 2012). The currently accepted genera are listed in Table 1.

**TABLE 1:** The current classification of the *Erysiphaceae.*

*ERYSIPHACEAE*
**Tribe *Erysipheae***
One genus: *Erysiphe*
**Tribe *Golovinomyceteae***
Subtribe *Neoerysiphinae*
One genus: *Neoerysiphe*
Subtribe *Golovinomycetinae*
One genus: *Golovinomyces*
Subtribe *Arthrocladiellinae*
One genus: *Arthrocladiella*
**Tribe *Cystotheceae***
Subtribe *Cystothecinae*
Two genera: *Cystotheca, Podosphaera*
Subtribe *Sawadaeinae*
One genus: *Sawadaea*
**Tribe *Phyllactinieae***
Four genera: *Leveillula, Phyllactinia, Pleochaeta, Queirozia*
**Tribe *Blumerieae***
One genus: *Blumeria*
**Tribe Unnamed**
One genus: *Microidium*
**Genera not included in the tribe-level classification:**
*Brasiliomyces, Bulbomicroidium, Caespitotheca, Parauncinula, Salmonomyces, Takamatsuella*

The nrDNA phylogenies have also led to the development of a new hypothesis about the evolution of the distinctive chasmothecial appendages of powdery mildews, and a better understanding of their life cycles. This was needed because, in contrast to the early speculations, all phylogenetic analyses have shown that species with complex appendages on their chasmothecia have appeared first, and those with simple, mycelioid appendages only later during evolution (for reviews, see Takamatsu, 2013a; Takamatsu, 2018). Most of the latter species infect herbaceous plants, while species with complex appendages are pathogens of trees or shrubs, and usually have more restricted host ranges than herb-pathogenic species (Mori et al., 2000a; Takamatsu et al., 2000; Takamatsu, 2013b). To explain these results, Takamatsu (2004) hypothesized that appendage morphology reflects the adaptation of the respective powdery mildew species to overwintering on deciduous woody hosts, mostly in regions with temperate climate. Appendages with complex geometries play a vital role in securing the attachment of many chasmothecia to the bark of their deciduous hosts at the end of the growing season, when those are washed off the canopy by autumn rains, or detached, e.g., by air movements, before leaf fall (for a review, see Takamatsu, 2013b). Some of the chasmothecia that become attached to the woody parts of their deciduous hosts survive the winter period, and release ascospores in spring in the close vicinity of the freshly emerged shoots and leaves, which are then infected by ascospores, re-starting the infection cycle. This was well documented for example in *Erysiphe necator* on grapevine (Pearson and Gadoury, 1987; Gadoury et al., 2012) and *E. alphitoides* on sessile oak (*Quercus petraea*) (Dantec et al., 2015). Takamatsu (2004) has also hypothesized that simple, mycelioid appendages of chasmothecia of diverse herb-parasitic powdery mildew fungi may be a result of convergent evolution that has repeatedly occurred in different lineages of the *Erysiphaceae* as an adaptation to their herbaceous host plants. It appears that evolutionary changes in the characteristics of the conidiogenesis (e.g., whether conidia are produced singly or in chains), reflected by the morphology of the conidiophores, have happened slower than changes in the morphology of chasmothecia, and nrDNA phylogenies of the *Erysiphaceae* are capturing their phenotypic evolution based on patterns of their conidiogenesis (Takamatsu, 2013b).

DNA regions other than nrDNA loci have also been tested for their use in delimiting species in some groups of the *Erysiphaceae*. The main objectives were to introduce new loci, including protein-coding regions, in phylogenetic analyses (Inuma et al., 2007; Liu et al., 2021); to develop new species-specific DNA barcodes (Ellingham et al., 2019; Shirouzu et al., 2020); and to test the hypothesis of interspecific hybridization in powdery mildew fungi (Seko et al., 2011). Intraspecific, host-driven differentiation of some powdery mildew species has also been tested with new DNA markers, e.g., in *Podosphaera xanthii* (Vela-Gorcía et al., 2014) and *Erysiphe quercicola* (Desprez-Loustau et al., 2017). Amongst the non-nrDNA loci, the *MCM7* gene was the most useful in distinguishing closely related powdery mildew taxa (Ellingham et al., 2019; Shirouzu et al., 2020). This gene encodes one of the highly conserved mini-chromosome maintenance proteins (MCMs) that is required for eukaryotic genome replication (Raja et al., 2011).

Internal transcribed spacer sequences are still the most commonly used species-level DNA barcodes available for powdery mildew fungi, despite their limitations. These include intragenomic variations within some powdery mildew species (Kovács et al., 2011) and lack of resolution power to discriminate between some other species that can be distinguished based on morphology and host range (Braun et al., 2019; Qiu et al., 2020). ITS sequences as species barcodes have limitations in other fungal groups as well (Kiss, 2012; Stadler et al., 2020).

Traditionally, powdery mildew fungi were classified as members of the monotypic order *Erysiphales* (i.e., consisting of a single family, the *Erysiphaceae*) (Braun and Cook, 2012). However, comprehensive multi-gene analyses of the class *Leotiomycetes* have recently shown that powdery mildew fungi group together with a few *Arachnopeziza* species within the order *Helotiales*, and together form the ‘erysiphoid clade’ (Johnston et al., 2019). A taxonomic consequence of this phylogenetic analysis is that the family level classification of powdery mildew fungi, i.e., the status of the *Erysiphaceae*, remains unchanged, but the order *Erysiphales* is not retained and the *Erysiphaceae* is now regarded to be part of the *Helotiales* sensu Johnston et al. (2019).

Another outcome of the multi-gene analyses performed by Johnston et al. (2019) is that *Arachnopeziza* spp. were identified as the closest known saprobic relatives of powdery mildew fungi. Little is known about *Arachnopeziza* spp.; it appears that these fungi are decomposers of diverse organic substrates, such as decaying wood and leaf debris, mostly in forest ecosystems (Hirata et al., 2000; Baldrian et al., 2016; Kosonen et al., 2021). Earlier, when the available nrDNA and other sequences were much more limited, the *Myxotrichaceae*, including *Oidiodendron*, *Byssoascus*, and *Myxotrichum* species were considered as the closest known saprobic relatives of the *Erysiphaceae* based on nrDNA phylogenies and molecular clock calculations (Berbee and Taylor, 1993, 2001; Sugiyama et al., 1999; Mori et al., 2000b; Takamatsu, 2004). Fungi belonging to the *Myxotrichaceae* are also known as decomposers of dead plant materials and other organic materials. Further phylogenetic analyses indicated that *Chlorociboria* and *Cyttaria* (Wang et al., 2006a,b) or the *Myxotrichaceae* and *Pleuroascus* (Peterson and Pfister, 2010) are the closest known saprobic relatives of powdery mildew fungi. Deciphering the evolutionary origin of the *Erysiphaceae* may shed light on how a fungal lineage has evolved from a saprobic lifestyle to obligate biotrophy.

A part of the comprehensive phylogenetic analyses performed by Johnston et al. (2019) used over 3,000 concatenated orthologous single-copy genes extracted from the genomes of 49 strains belonging to the *Leotiomycetes*. Their analysis showed that *Oidiodendron maius*, representing the *Myxotrichaceae*, was not a close relative of the ‘erysiphoid clade’ consisting of powdery mildew fungi and *Arachnopeziza*. This was also revealed by other analyses of the same study (Johnston et al., 2019) based on 15 genes commonly used in recent fungal phylogenies. Another genome-scale study revealed the phylogeny of the entire phylum *Ascomycota*, using 815 single-copy orthologs from genomes of 1,107 ascomycetes (Shen et al., 2020). That study did not include *Arachnopeziza* genomes but confirmed that *O. maius* is not closely related to powdery mildew fungi.

So far, only one study has reconstructed evolutionary relationships within the *Erysiphaceae* based on genome-scale data (Frantzeskakis et al., 2019a). The aim of that analysis was to test the relationship between *Parauncinula polyspora*, an early-diverged powdery mildew fungus with a surprisingly small genome, and other, well-known powdery mildews based on non-nrDNA loci. The analysis used 1,964 single-copy orthologs identified in the genomes of seven powdery mildew and nine other fungi belonging to the *Leotiomycetes*. *Arachnopeziza* genomes were not included in that study (Frantzeskakis et al., 2019a).

Clearly, further comprehensive genome-scale phylogenies are needed to learn more about the evolution of powdery mildews and to reveal their closest saprobic relatives. Comparative genomics analyses have already contributed to a better understanding of the interactions between some powdery mildew fungi and their plant hosts, including rapid evolution of the host range patterns (Spanu et al., 2010; Wicker et al., 2013; Frantzeskakis et al., 2018, 2019b; Wu et al., 2018; Barsoum et al., 2019; Müller et al., 2019), but comprehensive analyses of host-parasite interactions in many species and genera of the *Erysiphaceae* have not been performed yet. Phylogenetic analyses of nrDNA loci have already revealed many aspects of their evolution that are consistent with morphological characteristics of different groups, and their host-pathogen interaction patterns (Takamatsu, 2013a,b). Multi-gene studies may be useful to enrich results based solely on nrDNA analyses, above all to disentangle complexes of closely allied species that cannot be properly resolved in phylogenetic analyses based on ITS sequences only, such as the *Erysiphe aquilegiae* complex (Shin et al., 2019; Bradshaw et al., 2020), and to identify the closest saprobic relatives of the *Erysiphaceae*.

More and more genome assemblies are reported for different powdery mildew species (e.g., Kusch et al., 2020, 2022a; Kim et al., 2021; Polonio et al., 2021), and these data offer new avenues to understand the powdery mildew lifestyle. However, most powdery mildew genomes published so far are highly fragmented (Bindschedler et al., 2016; Barsoum et al., 2019) as these are generally large, gene-poor, and contain a high proportion of repetitive elements compared to other ascomycetes (Spanu et al., 2010; Wicker et al., 2013; Frantzeskakis et al., 2018). These quality issues sometimes limit the use of powdery mildew genomes in further studies. The main goals of this study were to (i) perform genome-scale phylogenetic analyses using single-copy orthologs identified in the genomes of as many powdery mildew fungi and presumed saprobic relatives as possible; (ii) compare the results to nrDNA phylogenies of the same isolates; (iii) reveal quality issues associated with the use of the currently available powdery mildew whole-genome sequencing (WGS) datasets in phylogenetic studies; and (iv) update the taxonomy of the genome-sequenced powdery mildew species where needed.

## Materials and Methods

### Genome-Scale Phylogenetic Analyses

To obtain a comprehensive set of genomes representative of powdery mildews sequenced to date, the European Nucleotide Archive (ENA), Joint Genome Institute (JGI), and National Center for Biotechnology Information (NCBI) were searched using the search term *Erysiphaceae* in January 2022. For *Blumeria graminis*, the most studied powdery mildew species infecting several cereal and wild grass species, and *Erysiphe necator*, the causal agent of grape powdery mildew, multiple genomes are available in public repositories; therefore, only four to five representative genomes were retrieved. For all other powdery mildew species that had a sequenced genome, all available genomes were obtained from the databases listed above, except for *E. alphitoides* specimen MS-42D (Dutech et al., 2020), for which only the raw data are available in ENA, and the genome was retrieved from http://arachne.pierroton.inra.fr/AlphiGeno/ (Table 2).

**TABLE 2:** List of powdery mildew genomes assessed for completeness through identification of Benchmarking Universal Single-Copy Orthologs (BUSCO) using BUSCO v.5.2.2 (Simão et al., 2015) and Leotiomycetes dataset Odb10.

Powdery mildew species	Isolate/specimen ID	Reference*^a^*	BUSCO results*^b^*				
			Genome completeness (%)	CS	CD	F	M
*Blumeria hordei* (formerly *B. graminis* f. sp. *hordei*)	A6	Hacquard et al., 2013	84.7	2,728	11	193	302
	**DH14**	Frantzeskakis et al., 2018	94.7	3,042	21	74	97
	K1	Hacquard et al., 2013	94.3	3,023	25	77	109
	**RACE1**	Frantzeskakis et al., 2018	94.6	3,041	21	73	99
*B. graminis* f. sp. *triticale*	**THUN-12**	Müller et al., 2021	94.8	3,043	22	69	100
*B. graminis* f. sp. *tritici*	**70**	Wicker et al., 2013	94.0	3,035	7	85	107
	94202	Wicker et al., 2013	93.3	3,007	10	103	114
	**96224**	Wicker et al., 2013; Müller et al., 2019	94.5	3,038	18	70	108
	JIW2	Wicker et al., 2013	91.9	2,963	9	124	138
*Erysiphe alphitoides*	**MS-42D**	Dutech et al., 2020	95.4	2,322	762	60	90
*E. necator*	**C**	Jones et al., 2014	93.4	3,017	2	80	135
	**e1-101**	Jones et al., 2014	93.3	3,015	2	83	134
	**Lodi**	Jones et al., 2014	93.2	3,014	1	82	137
	Ranch9	Jones et al., 2014	93.0	3,009	2	84	139
*E. neolycopersici* (formerly *Oidium neolycopersici*)	**UMSG2**	Wu et al., 2018	93.0	3,000	6	90	138
*E. pisi*	**Palampur-1**	Unpublished JGI	92.4	2,939	47	107	141
	–	Unpublished, NCBI Acc. GCA_000208805.1	56.2	1,818	1	550	865
	–	Unpublished, NCBI Acc. GCA_000214055.1	63.8	2,064	1	464	705
*Erysiphe pulchra*	**TENN-F-071826**	Wadl et al., 2019	91.7	2,950	17	130	137
*E. quercicola* (formerly *Oidium heveae*)	**HO-73**	Liang et al., 2018	92.8	2,990	10	100	134
*Golovinomyces cichoracearum*	**UCSC1**	Wu et al., 2018	91.1	2,926	18	76	214
	**UMSG3**	Wu et al., 2018	91.3	2,932	20	76	206
*G. magnicellulatus*	**FPH2017-1**	Farinas et al., 2019	92.2	2,692	290	71	181
*G. orontii*	MGH1	Unpublished, JGI Genome MGH1 v4.0	89.9	648	2,261	103	222
*Leveillula taurica*	**HNHM-MYC-006405**	Kusch et al., 2020	82.4	2,661	3	83	487
*Parauncinula polyspora*	**–**	Frantzeskakis et al., 2019a	93.0	2,991	15	27	201
*Phyllactinia moricola*	**HMJAU-PM91933**	Kusch et al., 2022b	71.3	2,306	0	236	692
*Pleochaeta shiraiana*	**HAL3440 F**	Kusch et al., 2022b	76.6	2,465	12	93	664
*Podosphaera cerasi*	**MH**	Unpublished, NCBI Acc. GCA_018398735.1	91.9	2,962	9	87	176
*P. leucotricha*	**PuE-3**	Gañán et al., 2020	91.6	2,952	9	99	174
*P. xanthii*	**2086**	Polonio et al., 2021	90.9	2,899	41	67	227
	**Wanju2017**	Kim et al., 2021	91.6	2,811	151	66	206

*Genomes of the isolates/specimens that were selected for the genome-scale phylogenetic analysis are indicated in bold.*

*^a^Where a reference for a genome assembly was not available, the genome accession number in National Centre for Biotechnology Information (NCBI) or Joint Genome Institute (JGI) is provided.*

*^b^Benchmarking Universal Single-Copy Orthologs. CS, complete and single copy BUSCOs; CD, complete and duplicated BUSCOs; F, fragmented BUSCOs; M, missing BUSCOs.*

To assess the quality of the accessed genome assemblies, we used the Benchmarking Universal Single-Copy Orthologs (BUSCO) v.2.5.5 (Simão et al., 2015) and the Leotiomycetes odb10 database, which includes a total of 3,234 single-copy BUSCOs. Where multiple genomes were available for a species, and to remove low-quality genomes and minimize missing data, only genomes with highest completeness were retained for the phylogenetic analyses. The final dataset for genome-scale phylogenetic analyses included 24 powdery mildew genomes (Table 2). A set of genomes selected to represent close relatives of powdery mildew fungi within the *Leotiomycetes* were also included in the genome-scale analysis (Table 3); these were selected based on previous phylum-level and class-level phylogenies (Johnston et al., 2019).

**TABLE 3:** List of non-powdery mildew Leotiomycete genomes included in the genome-scale phylogenetic analysis.

Species	Strain	Accession number	Database	References
*Amorphotheca resinae*	ATCC 22711	GCA_003019875.1	NCBI	Martino et al., 2018
*Amorphotheca resinae*	KUC3009	GCA_018167515.1	NCBI	Oh et al., 2021
*Arachnopeziza araneosa*	ICMP 21731	GCA_003988855.1	NCBI	Unpublished
*Ascocoryne sarcoides*	NRRL 50072	GCA_000328965.1	NCBI	Gianoulis et al., 2012
*Chlorociboria aeruginascens*	DSM 107184	GCA_002276475.2	NCBI	Büttner et al., 2019
*Glarea lozoyensis*	ATCC 20868	GCA_000409485.1	NCBI	Chen et al., 2013
*Marssonina brunnea* f. sp. *multigermtubi*	MB_m1	GCA_000298775.1	NCBI	Zhu et al., 2012
*Neobulgaria alba*	ICMP 18395	GCA_003988965.1	NCBI	Unpublished
*Oidiodendron maius*	Zn	GCA_000827325.1	NCBI	Kohler et al., 2015
*Phialocephala scopiformis*	CBS 120377	GCF_001500285.1	NCBI	Walker et al., 2016
*Phialocephala subalpine*	UAMH 11012	GCA_900073065.1	NCBI	Schlegel et al., 2016
*Rhynchosporium commune*	UK7	GCA_900074885.1	NCBI	Penselin et al., 2016
*Rhynchosporium secalis*	02CH4-6a.1	GCA_900074895.1	NCBI	Penselin et al., 2016
*Sclerotinia trifoliorum*	SwB9	GCA_905066765.1	NCBI	Kusch et al., 2022a

We used single-copy orthologous amino acid sequences obtained using BUSCO for phylogenomic inference. First, extracted protein sequences for all genomes were analyzed using OrthoFinder v.2.5.1 (Emms and Kelly, 2019) to identify single-copy orthologs shared across all genomes. OrthoFinder assigned a total of 113,072 proteins (99.9% of total) to 3,427 orthogroups and identified 751 orthogroups that existed in all target genomes in single copies. Amino acid sequences were aligned separately using MAFFT v.7.453 (Katoh and Standley, 2013) with the BLOSUM62 matrix of substitutions. Ambiguously aligned regions were removed using Gblocks v. 0.91b (Castresana, 2000; Talavera and Castresana, 2007) using default settings. A maximum likelihood (ML) phylogenetic tree based on the concatenated alignment of amino acid sequences was generated with 1,000 bootstrap replicates using RAxML-NG v.1.0.1 (Kozlov et al., 2019), under the JTT+I+G4+F amino acid substitution model identified by ModelTest-NG v.0.1.6 (Darriba et al., 2020). *Sclerotinia trifoliorum* strain SwB9 was used as the outgroup (Kusch et al., 2022b).

### Nuclear Ribosomal DNA Sequences From Whole-Genome Sequencing Datasets Versus Sanger Sequencing

We aimed to produce a separate phylogeny based on nrDNA sequences of the same set of powdery mildew specimens to compare with the phylogeny based on the single-copy orthologs. For this, we searched the NCBI GenBank database for 5.8S, 18S, and 28S nrDNA sequences of each powdery mildew specimen included in our genome-scale study (Table 4). Out of the 24 specimens included in the genome-scale analyses, only 10 had their nrDNA loci sequenced by Sanger sequencing, and only one of them, originally recognized as *Oidium heveae* HO-73, had all the three loci sequenced prior to this study (Table 4). (HO-73 should be identified as an isolate of *E. quercicola* based on Wu et al., 2019; see below.) Subsequently, we attempted to extract the missing nrDNA loci from the published genome assemblies for inclusion in the nrDNA phylogenetic analysis. For this, we converted the genome assemblies of target species to BLAST databases in Geneious Prime^1^ (Kearse et al., 2012) and used 5.8S, 18S, and 28S sequences of reference specimens (Kiss et al., 2020) as queries in BLAST searches against the genomes to retrieve the respective sequences from each of the assemblies. The extracted nrDNA fragments were used in BLAST searches against the NCBI nrDNA database to ensure these belonged to the target powdery mildew species and are suitable for the phylogenetic analysis. Where nrDNA sequences of specimens were available in the NCBI GenBank database, we aligned these against the sequences obtained from the genomes for comparison (Table 5). To avoid misidentification of sequences as nrDNA, the Megablast function was used, and contigs/scaffolds were only reported to contain nrDNA sequence fragments if these had a query coverage of 100% and BLAST hit length of at least 300 bp. The identified fragments were subsequently used in BLAST searches against the NCBI nrDNA database to ensure these belonged to the nrDNA region.

**TABLE 4:** List of powdery mildew specimens included in the genome-scale and nuclear ribosomal (nrDNA) phylogenetic analyses.

Powdery mildew species	Isolate/specimen ID	GenBank assembly accession	Database*^a^*	nrDNA accession no.		
				28S	5.8S	18S
*Blumeria hordei* (formerly *B. graminis* f. sp. *hordei*)	DH14	GCA_900239735.1	NCBI	OENG01000016.1 *^b^*	OENG01000016.1 *^b^*	OENG01000016.1 *^b^*
	RACE1	GCA_900237765.1	NCBI	UNSH01000070.1 *^c^*	UNSH01000070.1 *^c^*	UNSH01000070.1 *^c^*
*B. graminis* f. sp. *triticale*	THUN-12	GCA_905067625.1	NCBI	CAJHIT010000009.1 *^d^*	CAJHIT010000009.1 *^d^*	CAJHIT010000009.1 *^d^*
*B. graminis* f. sp. *tritici*	70	GCA_000441875.1	NCBI	ASJN01035784.1	ASJN01035784.1	ASJN01035784.1
	96224	GCA_900519115.1	NCBI	LR026992.1 *^e^*	LR026992.1 *^e^*	LR026992.1 *^e^*
*Erysiphe alphitoides*	MS_42D	–	–*^f^*	Contig 84	Contig 84	Contig 84
*E. necator*	C	GCA_000798715.1	NCBI	JNVN01000032 *^h^*	JNVN01000032 *^h^*	JNVN01000032 *^h^*
	e1-101	GCA_000798795.1	NCBI	JOKO01000133	JOKO01000133	JOKO01000133
	Lodi	GCA_000798775.1	NCBI	JNUU01000055	JNUU01000055	JNUU01000055
*E. neolycopersici* (formerly *Oidium neolycopersici*)	UMSG2	GCA_003610855.1	NCBI	n.d.*^i^*	**KX776199**	**KX776199**
*E. pisi*	Palampur-1	–	JGI	scaffold_125 *^j^*	scaffold_125 *^j^*	scaffold_125 *^j^*
*E. pulchra*	TENN-F-071826	GCA_002918395.1	NCBI	PEDP01018202.1 *^k^*	**MH766898**	n.d.*^i^*
*E. quercicola* (formerly *Oidium heveae*)	HO-73	GCA_003957845.1	NCBI	**KJ868175** *^g^*	**KJ868176** *^g^*	**KP171512** *^g^*
*Golovinomyces cichoracearum*	UCSC1	GCA_003611215.1	NCBI	n.d.*^i^*	**AF031282**	**AF031282**
	UMSG3	GCA_003611195.1	NCBI	n.d.*^i^*	**KR611314**	**KR611314**
*G. magnicellulatus*	FPH2017-1	GCA_006912115.1	NCBI	n.d.*^i,l^*	n.d.*^i,l^*	n.d.*^i,l^*
*Leveillula taurica*	HNHM-MYC-006405	PRJEB36538	ENA	OM906815*	**MT125856**	OM906851*
*Parauncinula polyspora*	–	PRJEB29715	ENA	OM906816*	OM906197*	OM906852*
*Phyllactinia moricola*	HMJAU-PM91933	GCA_019455665.1	NCBI	**MZ540403**	**MZ541088**	JAHYSQ010042711.1 *^m^*
*Pleochaeta shiraiana*	HAL3440 F	GCA_019455505.1	NCBI	OM906817*	**MZ661116**	OM906853*
*Podosphaera cerasi*	MH	GCA_018398735.1	NCBI	JAGTUB010000840.1 *^n^*	JAGTUB010000840.1 *^n^*	JAGTUB010000840.1 *^n^*
*Po. leucotricha*	PuE-3	GCA_013170925.1	NCBI	n.d.*^i^*	**MT180425**	n.d.*^i^*
*Po. xanthii*	2086	GCA_014884795.1	NCBI	**MK225554**	JACSEY010000300	**MK225523**
	Wanju2017	GCA_010015925.1	NCBI	JAAAXZ010001060	JAAAXZ010001060	JAAAXZ010001060

If available as a result of Sanger sequencing, 28S, 5.8S and 18S nrDNA sequences were obtained from the NCBI GenBank nucleotide database (accession numbers shown in **bold**). Some loci were determined by Sanger sequencing in this work (indicated by asterisk, *). If nrDNA sequences of some specimens were not available in the nucleotide database of GenBank, and could not be determined in this study, sequences of the corresponding regions were extracted from the published genomes (contig/scaffold numbers harboring the respective nrDNA regions underlined).

*^a^ENA, European Nucleotide Archive; JGI, Joint Genome Institute; and NCBI, National Centre for Biotechnology Information.*

*^b^Identical copies of nrDNA sequences were also detected in contig OENG01000318.1.*

*^c^Identical copies of nrDNA sequences were also detected in contigs UNSH01000068.1 and UNSH01000069.1.*

*^d^Identical copies of nrDNA sequences were also detected in contigs CAJHIT010000019.1, CAJHIT010000020.1, CAJHIT010000022.1, CAJHIT010000023.1, CAJHIT010000025.1, and CAJHIT010000030.1.*

*^e^Identical copies of nrDNA sequences were also detected in contig LR026995.1.*

*^f^Genome of Erysiphe alphitoides specimen MS-42D is available from http://arachne.pierroton.inra.fr/AlphiGeno/.*

*^g^Contigs QVIK01005055.1, QVIK01001208.1, QVIK01002109.1, and QVIK01007960.1 in the E. quercicola genome included nrDNA sequences, which were not included in the nrDNA analysis, as these were identical or highly similar to multiple other fungi and plant species.*

*^h^nrDNA sequences extracted from this contig showed high similarity to those of Erysiphe species, however, the 5.8S sequence was only partial, with 45 bp missing in the middle. Additional nrDNA sequences were detected in Contig JNVN01005878.1 in E. necator genome (GCA_000798715.1), which were identical to the nrDNA sequences of multiple Penicillium spp.*

*^i^n.d., not detected; the sequence of the corresponding region was not found in NCBI GenBank database or the published genome.*

*^j^Scaffold_34 also includes copies of nrDNA sequences that are identical or highly similar to those in Scaffold_125.*

*^k^This contig showed high similarity to 28S sequence of Erysiphe pulchra reference sequences on GenBank. Additional nrDNA sequences were detected in Contig PEDP01005487.1, which were identical to the nrDNA sequences of Cladosporium spp. Also, contig PEDP01002611.1 harbors partial 18S (609 bp) and 28S (1,291 bp) sequences identical and highly similar (99.8%) to those of Neohydatothrips annulipes.*

*^l^Contigs VCMJ01009734 and VCMJ01035023 in G. magnicellulatus genome harbor nrDNA sequences, which were not included in the analysis, as these showed high similarity to nrDNA sequences from Pseudozyma, Moesziomyces, and Acremonium spp.*

*^m^This contig included a very small fragment of 18S sequence (135 bp).*

*^n^Contig JAGTUB010000459.1 in Po. cerasi genome harbored nrDNA sequences identical to Aureobasidium pullulans and other ascomycetes.*

**TABLE 5:** Comparison of 28S, ITS and 18S sequences extracted from whole genome sequencing (WGS) datasets (genome assembly accession numbers shown in Table 4) to sequences determined by Sanger sequencing available at NCBI GenBank database.

Powdery mildew species	Isolate/specimen ID	Sanger sequencing (GenBank accession numbers)*^a^*			WGS (Contigs/scaffolds containing nrDNA sequence fragments)*^b^*			Number of nucleotide position differences*^c^*/alignment length		
				
		28S	ITS	18S	28S	ITS	18S	28S	ITS	18S
*Erysiphe quercicola* (formerly *Oidium heveae*)	HO-73	KJ868175	KJ868176	KP171512	QVIK010007 45.1*^d^*	QVIK01007 960.1*^e^*	QVIK010012 08.1*^f^*	0/328	–	–
					and QVIK010012 08.1*^f^*		and QVIK010021 09.1*^g^*	–	–	–
					and QVIK010050 55.1*^h^*		and QVIK010112 55.1*^i^*	–	–	–
*E. neolycopersici*	UMSG2	n.a.	KX776199	KX776199	n.d.*^j^*	n.d.	n.d.	–	–	–
*E. pulchra*	TENN-F-071826	n.a.	MH766898	n.a.	n.d.	PEDP01005487.1*^k^*	n.d.	–	–	–
*G. cichoracearum*	UCSC1	n.a.	AF031282	AF031282	n.d.	n.d.	n.d.	–	–	–
	UMSG3	n.a.	KR611314	KR611314	n.d.	n.d.	n.d.	–	–	–
*Leveillula taurica*	HNHM-MYC-006405	OM906815	MT125856	OM906851	scaffold_175 59*^l^*	scaffold_04895*^m^*	scaffold_04895*^m^*	–	41/585	894/1,797
							and scaffold_17559^*l*^	–	–	–
*Parauncinula polyspora*	-	OM906816	OM906197	OM906852	NODE_26095*^n^*	NODE_26095*^n^*	NODE_26095*^n^*	3/813	0/566	0/423
*Phyllactinia moricola*	HMJAU-PM91933	MZ540403	MZ541088	n.a.	JAHY SQ010042711.1	JAHYSQ010042711.1	n.d.	0/721	0/563	–
*Pleochaeta shiraiana*	HAL3440 F	OM906817	MZ661116	OM906853	JAHY SP010014424.1	JAHYSP010014424.1	JAHYSP010014424.1	0/241	0/445	0/1,736
							and JAHYSP010004388.1*^o^*	–	–	32/427
*Podosphaera leucotricha*	PuE-3	n.a.	MT180425	n.a.	n.d.	n.d.	n.d.	–	–	–
*Po. xanthii*	2086	MK225554	n.a.	MK225523	JACSEY010000300.1	n.d.	JACSEY010000300.1	28/3,019	–	4/899

*^a^n.a., not available.*

*^b^The nrDNA sequences obtained from NCBI GenBank database were used as BLAST queries to extract similar sequences from the corresponding genomes in Geneious Prime using Megablast. Contigs/scaffolds were only reported to contain nrDNA sequence fragments if they had a query coverage of 100% or BLAST hit length of at least 300 bp.*

*^c^Number of nucleotide positions with variable characters, i.e., positions with single nucleotide polymorphisms or indels, between nrDNA sequences from WGS versus Sanger sequencing. This information is provided only when the detected contig was identified as nrDNA fragments belonging to powdery mildew species. Dashes (‘–’) indicate that comparisons were meaningless, due to lack of data or because the nrDNA sequences did not come from the respective powdery mildew isolate.*

*^d^A 328 bp sequence extracted from this contig was identical to the 28S sequence of Oidium heveae (E. quercicola) (KJ868175).*

*^e^A 369 bp fragment from this contig was identical to the ITS region of Curvularia spp.*

*^f^Two fragments of 666 bp and 353 bp from this contig were highly similar (>98%) and identical to 28S and 18S sequences of multiple plant species.*

*^g^A 500 bp fragment from this contig showed high similarity (>99%) to species belonging to the class Arachnida.*

*^h^A 490 bp fragment from this contig showed high similarity (99.8%) to Cladosporium spp.*

*^i^This contig is 320 bp and identical to 18S sequence of multiple Cladosporium spp.*

*^j^n.d., not detected; the sequence of the corresponding region was not detected in the published genome.*

*^k^A 611 bp fragment from this contig is identical to the ITS sequence of multiple Cladosporium spp.*

*^l^Two 786 bp and 1,747 bp fragments from this contig were identical and highly similar (99.7%) to 28S and 18S sequences of multiple Penicillium spp.*

*^m^This contig harbored 18S and ITS sequences with similarity to Leveillula spp., however, large areas of dissimilarity were also identified (Figure 3).*

*^n^The Parauncinula polyspora contig NODE_26095, which harbors the nrDNA region (Frantzeskakis et al., 2019a), was missing from the genome that was retrieved from ENA and was obtained from Lamprinos Frantzeskakis (personal communication).*

*^o^This contig also included a partial 18S sequence with only 94% similarity to the 18S sequence of Pleochaeta shiraiana HAL3440 F.*

In addition, we amplified and sequenced the 18S and 28S regions in *Leveillula taurica* HNHM-MYC-006405 and *Pleochaeta shiraiana* HAL3440 F, as well as the 18S, 28S and ITS regions of the *P. polyspora* specimen studied by Frantzeskakis et al. (2019a), according to the protocol described by Kiss et al. (2020). The newly obtained nrDNA sequences were deposited in GenBank (Table 4).

### Nuclear Ribosomal DNA Phylogenetic Analysis

The 5.8S, 18S, and 28S sequences extracted from genome assemblies, retrieved from NCBI GenBank or determined in this work (Table 4), were included in the nrDNA analysis. When one or more of these loci were not available in either of the respective genomes or as separate GenBank entries for the respective specimens, and we did not have access to herbarium specimens or DNA from the respective specimens to amplify and sequence the loci, these were coded as missing. All powdery mildew specimens included in the genome-scale phylogenetic analysis were also used in the nrDNA-based phylogenetic analysis, except for *Golovinomyces magnicellulatus* FPH2017-1 as the nrDNA loci were missing from its genomes and were not available as separate entries in GenBank.

Sequences available for each region were aligned using MAFFT v.7.450 (Katoh and Standley, 2013) as implemented in Geneious Prime. Alignments were manually trimmed and concatenated into a supermatrix, with missing data represented as gaps. A Maximum Likelihood analysis of the concatenated alignment was run using RAxML v.8 (Stamatakis, 2018) in Geneious Prime with 1,000 bootstrap replicates based on the GTR substitution model with gamma-distribution rate variation for individual partitions. *Parauncinula polyspora* was used as the outgroup based on Kusch et al. (2022a).

### Nomenclature of the Genome-Sequenced Powdery Mildew Species

Some of the published powdery mildew genome assemblies are available in GenBank under species names that need to be updated. The taxonomy of the genus *Blumeria* has recently changed (Liu et al., 2021); we followed the new nomenclature and renamed the powdery mildew isolates from barley as *B. hordei* in this work. The species first described as *Oidium neolycopersici* from tomato (Kiss et al., 2001) was re-classified as *Pseudoidium neolycopersici* (Braun and Cook, 2012) and recently as *Erysiphe neolycopersici* (Hsiao et al., 2022). Therefore, the latter name was used here as a synonym of *O. neolycopersici*. Powdery mildew on rubber tree (*Hevea brasiliensis*) has long been attributed to *Oidium heveae*; however, Wu et al. (2019) revealed that the causal agent of this disease is *Erysiphe quercicola*, a species known to infect diverse host plant species. As the ITS sequence of the genome-sequenced isolate known as *O. heveae* HO-73, available in GenBank under acc. no. KJ868176, is identical to several ITS sequences of *E. quercicola* analyzed by Wu et al. (2019), we propose to use the binomial *E. quercicola* for isolate HO-73, especially because of confusions concerning the precise identification of powdery mildew anamorphs listed as *O. heveae* in different works (Braun and Cook, 2012; Wu et al., 2019).

## Results

### Genome-Scale Phylogenetic Analysis

**FIGURE 1:**
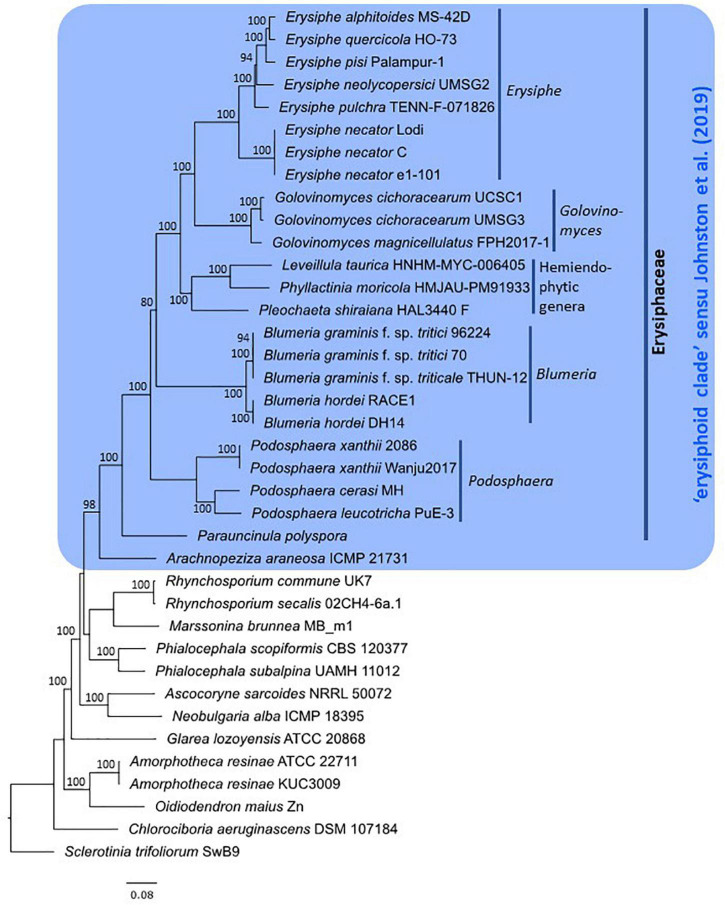
Phylogeny of powdery mildew species and closely related *Leotiomycetes* based on 751 orthologous protein sequences. Maximum likelihood phylogeny was inferred from a concatenated alignment of amino acid sequences using RAxML-NG v.1.0.1 (Kozlov et al., 2019) under the JTT+I+G4+F substitution model. Taxon labels include species names followed by the specimen/strain accession numbers, except for *Parauncinula polyspora*, for which a herbarium specimen is not available (Frantzeskakis et al., 2019a). Bootstrap support values greater than 70% are shown at the edges. The tree is rooted to *Sclerotinia trifoliorum* strain SwB9 (Kusch et al., 2022b). The scale bar represents nucleotide substitutions per site.

After assessment of publicly available powdery mildew genome assemblies, representative genomes of all powdery mildew species with published WGS data were included in the genome-scale phylogenetic analysis (Table 4) except for *Golovinomyces orontii* MGH1 (Micali et al., 2008) as its genome showed a high number of duplicated BUSCOs (2,261) and only 648 single-copy BUSCOs (Table 2). Therefore, the analysis was undertaken based on 751 single-copy orthologous sequences from 38 selected Leotiomycete genomes (24 powdery mildew genomes and 14 additional genomes from *Helotiales*) (Tables 3, 4). The final alignment included a total of 197,082 sites, with 0.74% gaps and 30.73% invariant sites. In the resulting phylogeny, the *Erysiphaceae* formed a monophyletic group with maximum bootstrap support as expected. *Arachnopeziza araneosa* was identified as the closest saprobic relative of powdery mildew fungi (Figure 1), which is in agreement with the analysis of Johnston et al. (2019). Alignments and trees produced in this study are available in the Supplementary Material.

The 24 powdery mildew genomes included in the analysis represented eight genera out of 19 recognized within the *Erysiphaceae* (Table 1). The epiphytic genera *Erysiphe, Golovinomyces, Blumeria*, and *Podosphaera*, all represented by multiple genomes, belonged each to distinct clades with 100% bootstrap support (Figure 1). Within *Erysiphe, E. alphitoides, E. quercicola*, and *E. pulchra* representing sect. *Microsphaera* (Takamatsu et al., 2015a), together with *E. pisi* belonging to sect. *Erysiphe*, formed a lineage with maximum bootstrap support. *Erysiphe necator*, a representative of sect. *Uncinula* within the genus (Takamatsu et al., 2015b), belonged to another lineage. Within *Podosphaera*, *P. leucotricha* and *P. cerasi*, representing sect. *Podosphaera* of the genus, formed a fully supported clade, while *P. xanthii*, a representative of sect. *Sphaerotheca*, belonged to another clade. Similar to a previous study (Frantzeskakis et al., 2019a), the analysis identified *Parauncinula polyspora*, another epiphytic powdery mildew species, as belonging to an early diverged lineage of the *Erysiphaceae*. Three genera, *Leveillula*, *Phyllactinia*, and *Pleochaeta*, each represented by a single genome, formed a distinct lineage known as the hemiendophytic lineage within the *Erysiphaceae* (Takamatsu, 2013a,b; Kusch et al., 2022a).

Out of those 14 other taxa from the *Helotiales* included in this analysis, *Arachnopeziza araneosa* was the only one that belonged to the large monophyletic clade including all the 24 genome-sequenced powdery mildew fungi (Figure 1). The close phylogenetic relationship between the *Erysiphaceae* and *Arachnopeziza* has already been shown by Johnston et al. (2019) based on the analysis of the class *Leotiomycetes* that included three powdery mildew species and was built on a partly overlapping set of a total of 3,156 single-copy orthologs.

### Nuclear Ribosomal DNA Phylogeny

**FIGURE 2:**
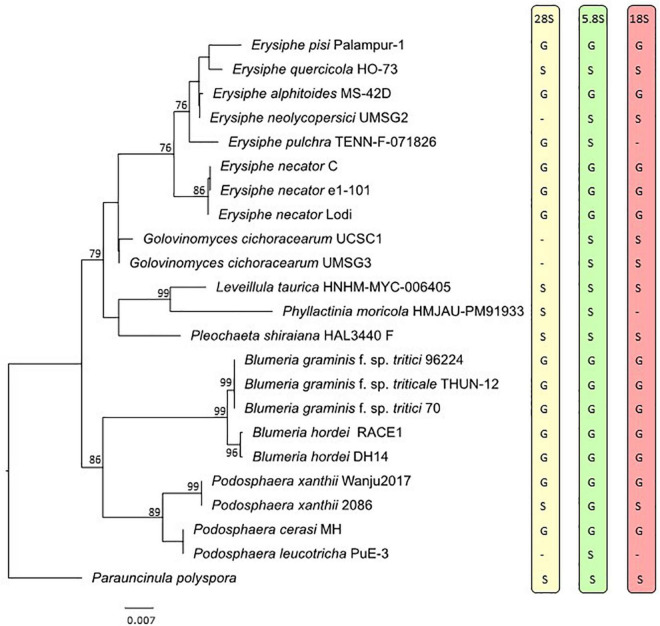
Maximum likelihood phylogeny based on the concatenated sequences of the 5.8S, 18S, and 28S regions of the nuclear ribosomal DNA of representative powdery mildew taxa. Bootstrap values greater than 70% are shown above or below the branches. The tree is rooted to *Parauncinula polyspora* specimen sequenced by Frantzeskakis et al. (2019a). Taxon labels include species names followed by the specimen accession numbers, except for *Parauncinula polyspora*, for which a herbarium specimen is not available (Frantzeskakis et al., 2019a). The letters on the right side of the tree indicate source of the 28S, 5.8S, and 18S nrDNA sequences as G (extracted from the genome) or S (produced through Sanger sequencing or obtained from NCBI GenBank database). Dashes indicate missing loci. The scale bar represents nucleotide substitutions per site.

All powdery mildew specimens included in the genome-scale phylogenetic analysis were also used in the nrDNA analysis, except for *G. magnicellulatus* FPH2017-1 as the 5.8S, 18S, and 28S nrDNA sequences were missing from its published genome. These sequences were not available for this specimen as separate entries in GenBank either and we had no access to any materials of the *G. magnicellulatus* isolate FPH2017-1 to determine the missing nrDNA sequences in this study. Therefore, the nrDNA analysis consisted of 23 taxa and a total of 2,666 sites (5.8S: 153 bp, 18S: 1,695 bp, and 28S: 818 bp), 34.8% of which were variable. Whenever possible, the 5.8S, 18S, and/or 28S nrDNA sequences extracted from the respective genomic databases were used in the analysis. When some of those sequences were not found in the respective WGS contigs/scaffolds, sequences from the same specimens/isolates were retrieved from GenBank or PCR-amplified and determined in this study or coded as missing. The nrDNA phylogeny (Figure 2) was largely congruent to the phylogeny produced based on 751 orthologous proteins (Figure 1).

### Quality of Nuclear Ribosomal DNA Sequences Extracted From Whole-Genome Sequencing Data Versus Sanger Sequencing

Extracting the nrDNA sequences from published powdery mildew genomes resulted in detection of contaminating DNA in multiple genomes (Table 4). For example, multiple nrDNA sequences were detected in the genome of *E. necator* C genome (GCA_000798715.1), which were identical to those of *Penicillium* spp. strains. Likewise, additional nrDNA sequences were detected in *E. pulchra* TENN-F-071826 (GCA_002918395.1) and *E. quercicola* (formerly *O. heveae*) HO-73 (GCA_003957845.1) genomes, which were highly similar to the nrDNA sequences of *Cladosporium* spp. and various plant species, respectively. Although no nrDNA sequences with any similarity to powdery mildew species could be retrieved from the genome of *G. magnicellulatus* (GCA_006912115.1), two contigs (VCMJ01009734 and VCMJ01035023) were found to carry nrDNA sequences with high similarity to nrDNA sequences of *Pseudozyma* spp., *Moesziomyces* spp., and *Acremonium* spp. (Table 4).

**FIGURE 3:**
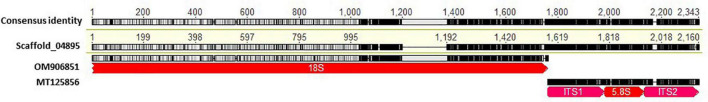
Alignment of 18S (GenBank accession no. OM906851) and ITS (GenBank accession no. MT125856) sequences of *Leveillula taurica* HNHM-MYC-006405 produced *via* Sanger sequencing against the sequence of Scaffold-04895 extracted from the genome assembly of the same strain (Assembly project PRJEB36538 from ENA database). nrDNA annotations are depicted by arrowed boxes. Black and gray colors indicate dissimilar and identical nucleotides, respectively, with gaps depicted by horizontal lines.

The presence of multiple dissimilar copies of nrDNA sequences in some powdery mildew assemblies prompted us to identify powdery mildew nrDNA sequences within the analyzed genomes and compare those to the 18S, 28S and ITS sequences of the same specimens if they were available in NCBI GenBank database. In some powdery mildew genome assemblies, nrDNA sequences obtained from Sanger sequencing and WGS data were identical (for example *Phyllactinia moricola* HMJAU-PM91933 and *Pleochaeta shiraiana* HAL3440 F; Table 5). In others, some or all the available nrDNA sequences differed in a few, or many, nucleotide positions when extracted from WGS contigs and compared to the results of Sanger sequencing (Table 5). As an example, Figure 3 reveals the variation of nrDNA sequences obtained by the two methods in *Leveillula taurica* HNHM-MYC-006405.

## Discussion

The nuclear ribosomal DNA region is an essential component of the genomes of all prokaryotes and eukaryotes as the genes included in this region encode ribosomal RNA (rRNA) molecules. These molecules are integral parts of cytoplasmic ribosomes, the major protein synthesis machinery of all living cells. All eukaryotic genomes contain multiple, tandemly repeated copies of a cluster consisting of 18S, 5.8S, and 28S rRNA genes, and the ITS1 and ITS2 regions flanking the 5.8S rRNA gene. This unit is sometimes designated as the 45S nrDNA cluster (Rosato et al., 2016) and it is first transcribed into a single precursor RNA, which is then further processed to produce the 18S, 5.8S and 28S rRNA molecules (Eickbush and Eickbush, 2007). The nuclear ribosomal 5S gene, which is another multi-copy nrDNA unit in all eukaryotic genomes, is transcribed independently of the 45S nrDNA cluster (Naidoo et al., 2013) and it may be localized in another part of the genome (Müller et al., 2019).

Multiple copies of both the 45S and the 5S nrDNA units are mostly needed in those stages of the cell cycles when the demand for protein synthesis is high. In fungi, a comprehensive genome-based analysis revealed that the copy number of the 45S nrDNA cluster varied considerably across phylogenetic lineages, ranging from an estimated 14 to 1,442 copies, with a mean value of 113 copies in the 91 taxa examined (Lofgren et al., 2019). Within the *Erysiphaceae*, *B. graminis* is the only species with an estimated copy number of the nrDNA units: Müller et al. (2019) detected approximately 800 copies of the 45S nrDNA cluster on chromosome 9 of *B. graminis*, and approximately 1,300 copies of the 5S nrDNA unit on chromosome 5.

There is usually low or no intragenomic variation amongst sequences of the nrDNA units, which has been attributed to the concerted evolution of these loci (Eickbush and Eickbush, 2007; Naidoo et al., 2013). In fungi, the 18S and 28S rRNA genes, and especially the ITS region that includes the 5.8S rRNA gene, have long been the most important and best-known DNA regions during phylogenetic and identification works (Schoch et al., 2012). In the present study it was, therefore, surprising to note how difficult is to work with the nrDNA region in some powdery mildew genomes. Some publicly available genome assemblies did not contain all rRNA genes of the nrDNA region (Figure 2 and Table 4). In two WGS assemblies, the 28S, ITS, and/or 18S nrDNA sequences differed in many nucleotide positions from those determined by Sanger sequencing in the same specimens (Figure 3 and Table 5). These differences may have been the result of mis-assembly issues that are common with short-read WGS. Conversely, all nrDNA loci were always reliably amplified through specific PCRs, and their sequences were accurately determined by Sanger sequencing in the *Erysiphaceae* (e.g., Marmolejo et al., 2018; Bradshaw and Tobin, 2020; Kiss et al., 2020). Therefore, we suggest that the presence of 28S, ITS, and 18S rDNA sequences in powdery mildew (and other) WGS datasets that are identical to those resulted from Sanger sequencing of the respective loci should be used to assess the quality of assemblies, in addition to the commonly used BUSCO values.

Our study has also revealed that a number of published powdery mildew genome assemblies are contaminated with nrDNA sequences from non-target organisms. Misclassification of sequences in reference databases and contamination of public genome assemblies with sequences from other organisms is a common problem that has been the subject of many studies (Kryukov and Imanishi, 2016; Breitwieser et al., 2019; Lupo et al., 2021). Our analyses indicated that contaminant sequences are common in some of the draft powdery mildew genomes, which are, in fact, metagenomes. This is partly linked to the obligate biotrophic nature of powdery mildews, i.e., the fact that isolates cannot be grown in the absence of their host plants. DNA has to be extracted from powdery mildew samples that almost inevitably contain host plant DNA and also DNA from multiple non-target organisms (predominantly microbes) that are associated with powdery mildew colonies (Panstruga and Kuhn, 2019). Contamination in genome assemblies can complicate downstream analyses and lead to misleading results; therefore, it is necessary to implement multiple methods and algorithms to assess and exclude contaminant sequences from draft powdery mildew and other genomes before making these public (Cornet et al., 2018; Kahlke and Ralph, 2018; Low et al., 2019; Wood et al., 2019; Kusch et al., 2020, 2022a).

Genome assemblies of other obligate biotrophic plant pathogens may also be contaminated with DNA regions coming from non-target organisms. For example, Zaccaron and Stergiopoulos (2021) have recently pointed out that the genome assembly of the oomycete *Albugo laibachii* infecting the leaves of experimental *Arabidopsis thaliana* plants (Kemen et al., 2011) contains the ITS region and many GC-rich regions of a powdery mildew fungus (*Golovinomyces* sp.), which may have infected the sampled leaves in addition to *A. laibachii* without being noticed.

The debate related to specimen-based versus environmental DNA (eDNA)-only research in biology, and particularly in fungal biology, is ongoing, especially in biodiversity and taxonomy studies (e.g., Truong et al., 2017; Lücking and Hawksworth, 2018). DNA samples of powdery mildew fungi used for WGS can be considered as eDNA because they are not extracted from pure cultures of the target isolates, as explained above. There is no consensus amongst diverse laboratories engaged in powdery mildew WGS to deposit herbarium specimens and/or other samples, such as DNA samples, of the powdery mildew-infected plant materials used for WGS in internationally recognized herbaria, fungaria or other fungal collections. This is the reason for the missing specimen or voucher accession numbers for some of the powdery mildew materials that were included in WGS projects and used in this study. Deposition of such materials in internationally accessible collections should be required by research journals before the publication of WGS results. The collections would preserve the specimens and other samples and would make them available for further studies similar to fungal biodiversity and taxonomy studies (Verkley et al., 2015).

This work included all powdery mildew species with publicly available genomes that were suitable for a genome-scale phylogenetic analysis using single-copy proteins. In total, 24 powdery mildew genome assemblies were used in the phylogenetic analyses, which represented eight out of the 19 genera that are currently recognized within the *Erysiphaceae*. Our analysis built on 751 single-copy orthologs resulted in a phylogeny that is largely congruent to nrDNA sequences-based phylogeny of the same set of specimens. These results indicated that phylogenetic analyses of nrDNA sequences are sufficient to delimit genera within the *Erysiphaceae*, which are also defined based on morphological characteristics (Braun and Cook, 2012; Marmolejo et al., 2018; Kiss et al., 2020). To test this presumption, further phylogenomic analyses should be conducted when genome assemblies become available for those powdery mildew genera that currently lack WGS data.

One of the main goals of this work was to reveal the closest known saprobic relatives of powdery mildew fungi. Recently, new genome assemblies were published for a number of taxa belonging to the order *Helotiales* (Table 3), which has enabled a more extensive taxon sampling and higher resolution of phylogenomic analyses within those fungal groups that were considered earlier as close saprobic relatives of the *Erysiphaceae*. Our genome-scale phylogenetic analysis identified the *Arachnopezizaceae* as a putative sister group of powdery mildew fungi. According to our study, the *Myxotrichaceae* (including *Oidiodendron*), which were previously considered as the closest saprobic relatives of powdery mildew fungi (Peterson and Pfister, 2010; Takamatsu, 2013a,^2018^), and *Chlorociboria*, another genus considered earlier as a close relative of the *Erysiphaceae* (Wang et al., 2006a,b), were only distantly related to powdery mildew fungi. Our results supported the finding of a comprehensive genome-scale analysis of 49 *Leotiomycetes* genomes that included the genomes of three powdery mildew species, and a genome of an *Arachnopeziza*, a *Chlorociboria*, and an *Oidiodendron* strain, and that grouped the three powdery mildew fungi and *Arachnopeziza* together in the newly defined ‘erysiphoid’ clade of the *Helotiales* (Johnston et al., 2019). Further analyses of powdery mildew and *Arachnopeziza* genomes may discover signatures of the evolutionary processes that have led to obligate biotrophy from a saprobic way of life.

## Data Availability Statement

DNA sequence data produced in this study were deposited in NCBI GenBank database and accession numbers are presented in Table 4. Alignments and phylogenetics trees are provided as Supplementary Material.

## Author Contributions

LK designed and coordinated the research. NV and LK wrote the manuscript. NV carried out all phylogenetic and phylogenomic analyses. SK contributed to genome analyses. MN and DS determined all 18S, ITS and 28S sequences newly reported in this study. UB provided taxonomic expertise, ST and RP phylogenetic and genomic expertise. UB, ST, RP, SK, and LK provided expertise on powdery mildew evolution and biology. All authors commented on early drafts of the manuscript, read, and approved the final version.

## Conflict of Interest

The authors declare that the research was conducted in the absence of any commercial or financial relationships that could be construed as a potential conflict of interest.

## Publisher’s Note

All claims expressed in this article are solely those of the authors and do not necessarily represent those of their affiliated organizations, or those of the publisher, the editors and the reviewers. Any product that may be evaluated in this article, or claim that may be made by its manufacturer, is not guaranteed or endorsed by the publisher.

## Footnotes

1
http://www.geneious.com
